# Supplementary material for: Characterization of a membrane-bound C-glucosyltransferase responsible for carminic acid biosynthesis in Dactylopius coccus Costa
Source: Nat Commun. 2017 Dec 7;8:1987. doi: 10.1038/s41467-017-02031-z (PMC5719414; doi:10.1038/s41467-017-02031-z)
Supplement: Supplementary file 1 — Supplementary Information [file 41467_2017_2031_MOESM1_ESM.pdf]

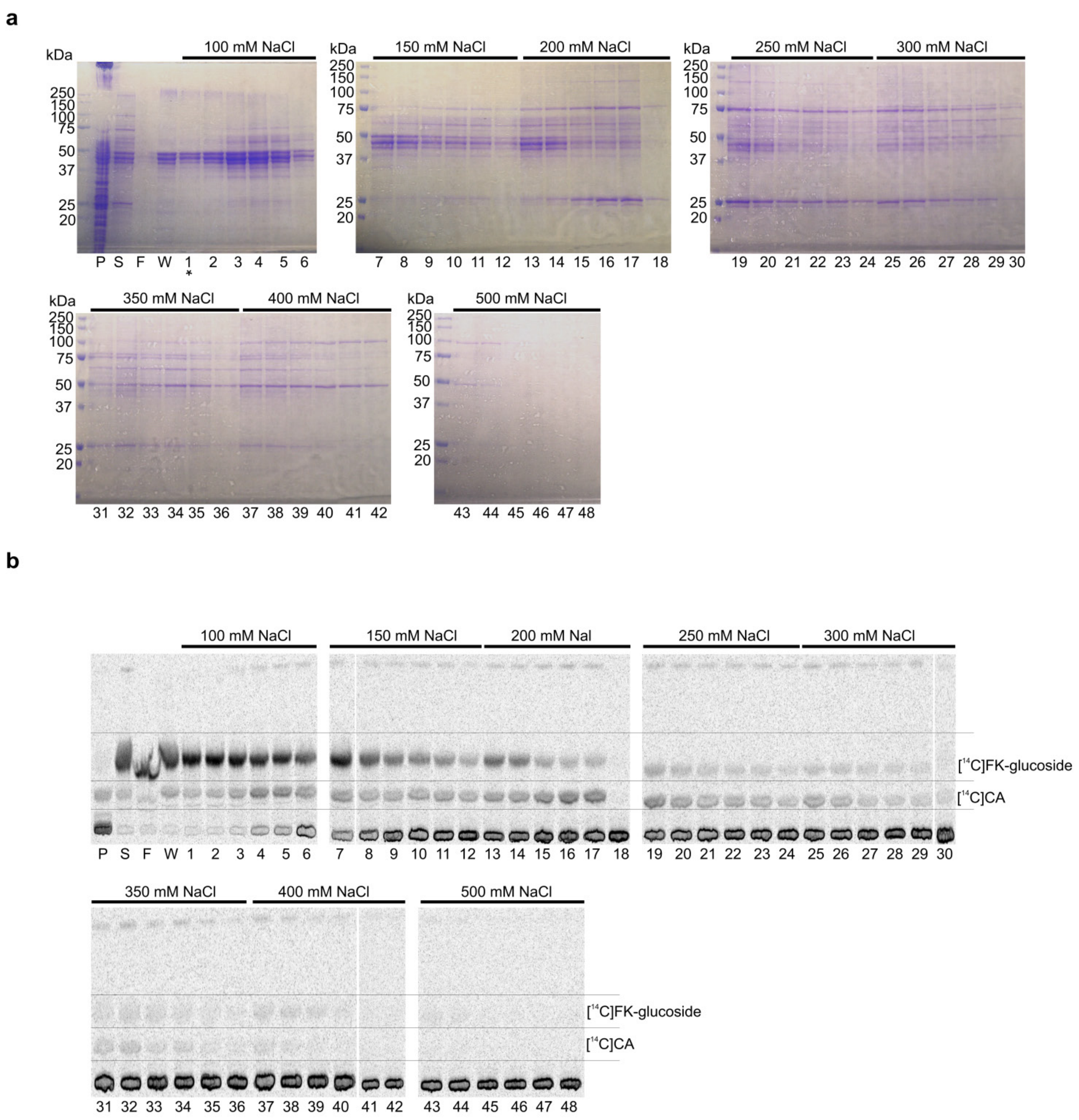

Supplementary Figure 1 | Partial purification of a *D. coccus* FK/KA-specific glucosylation activity *D. coccus* microsomes were solubilized using reduced Triton X-100 and fractionated by anion-exchange chromatography using Q-Sepharose. Proteins were eluted using a discontinues NaCl gradient from 100 mM-500 mM. Forty-eight eluted fractions were collected together with a pellet (P: Reduced Triton X-100 insoluble protein fraction), a solubilized (S: Reduced Triton X-100 soluble protein fraction), a flow-through (F: Proteins that did not bind to the Q-Sepharose) and a wash fraction (W: Proteins washed off the column with 50 mM NaCl). All protein fractions were tested for glucosylation activity *in vitro* using the flavokermesic acid aglucone and the [^{14}C]UDP-glucose donor. **a** Coomassie-stained SDS gels of separated protein fractions. **b** TLC-separated [^{14}C] products formed *in vitro* and viewed by phosphorimaging. FK: flavokermesic acid; CA: carminic acid; Asterisk indicates the protein fraction that was analyzed further by proteomics (Fig. 3). The positions of [^{14}C]FK-glucoside and [^{14}C]CA are indicated. The *in vitro* formation of [^{14}C]CA was ascribed to the conversion of kermesic acid that still was bound to the *D. coccus* microsomes despite numerous wash steps during preparation.

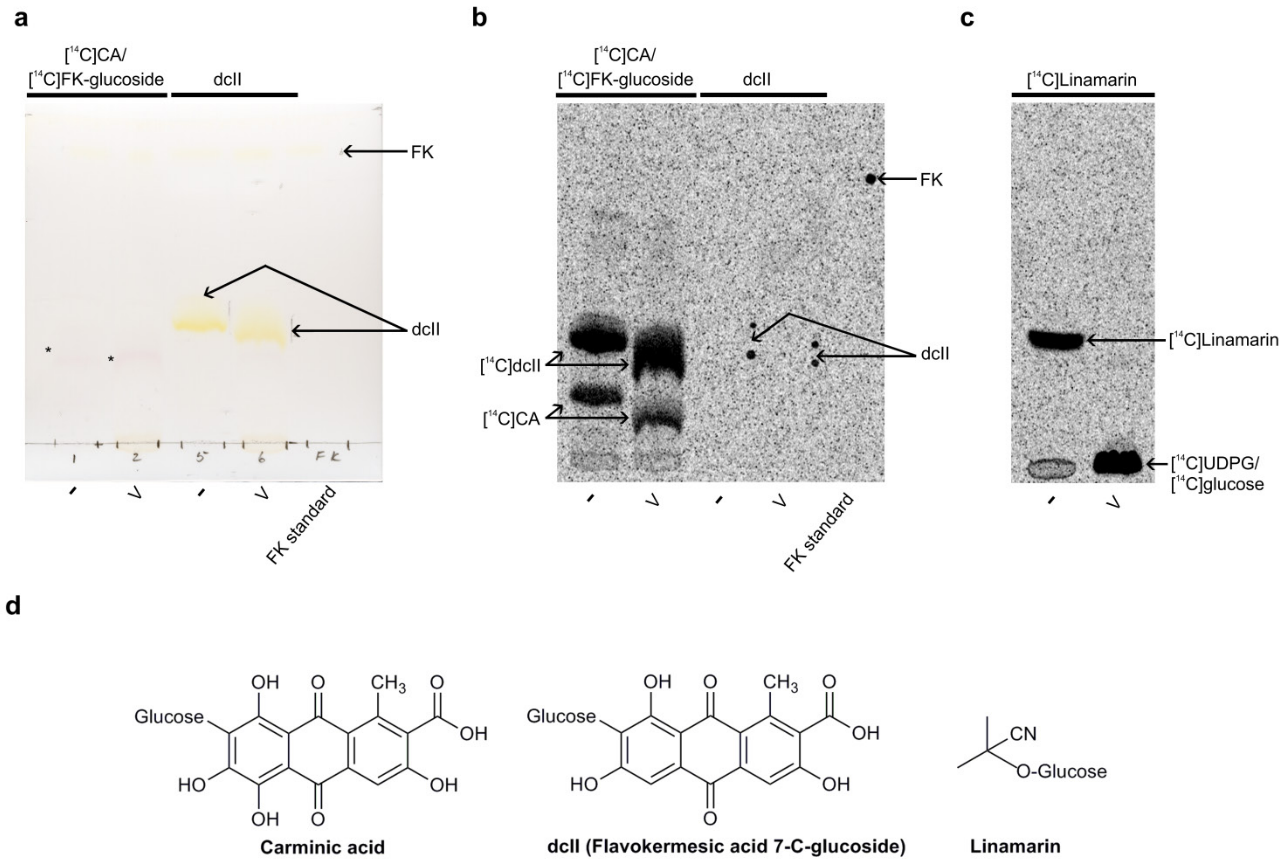

Supplementary Figure 2 | **Enzymatic verification of *in vitro*-produced dcII and carminic acid** [^{14}C] products, formed *in vitro* by incubating *D. coccus* microsomes with the flavokermesic acid aglucone and the [^{14}C]UDP-glucose donor, were isolated and either non-treated (-) or treated with viscozyme (V). Non-radioactive dcII and [^{14}C]Linamarin, representing a C-glucoside and an O-glucoside, respectively were used as controls for the viscozyme -treatment. **a** TLC plate of separated radioactive and non-radioactive compounds after treatment without or with viscozyme. **b** TLC plate from panel (**a**) viewed by phosphorimaging. **c** [^{14}C]Linamarin treated without or with viscozyme followed by TLC-separation and phosphorimaging. **d** Chemical structures of the glucosides treated without or with viscozyme. Asterisk indicates carminic acid that still was bound to the *D. coccus* microsomes despite numerous wash steps during preparation. Likewise, the *in vitro* formation of [^{14}C]CA was ascribed to the conversion of kermesic acid that also still was associated to the *D. coccus* microsomes. FK: flavokermesic acid; CA: carminic acid; [^{14}C]UDPG: [^{14}C]UDP-glucose. Note the slight aberrant product migration of viscozyme-treated samples caused by the increased viscosity from the viscozyme solution.

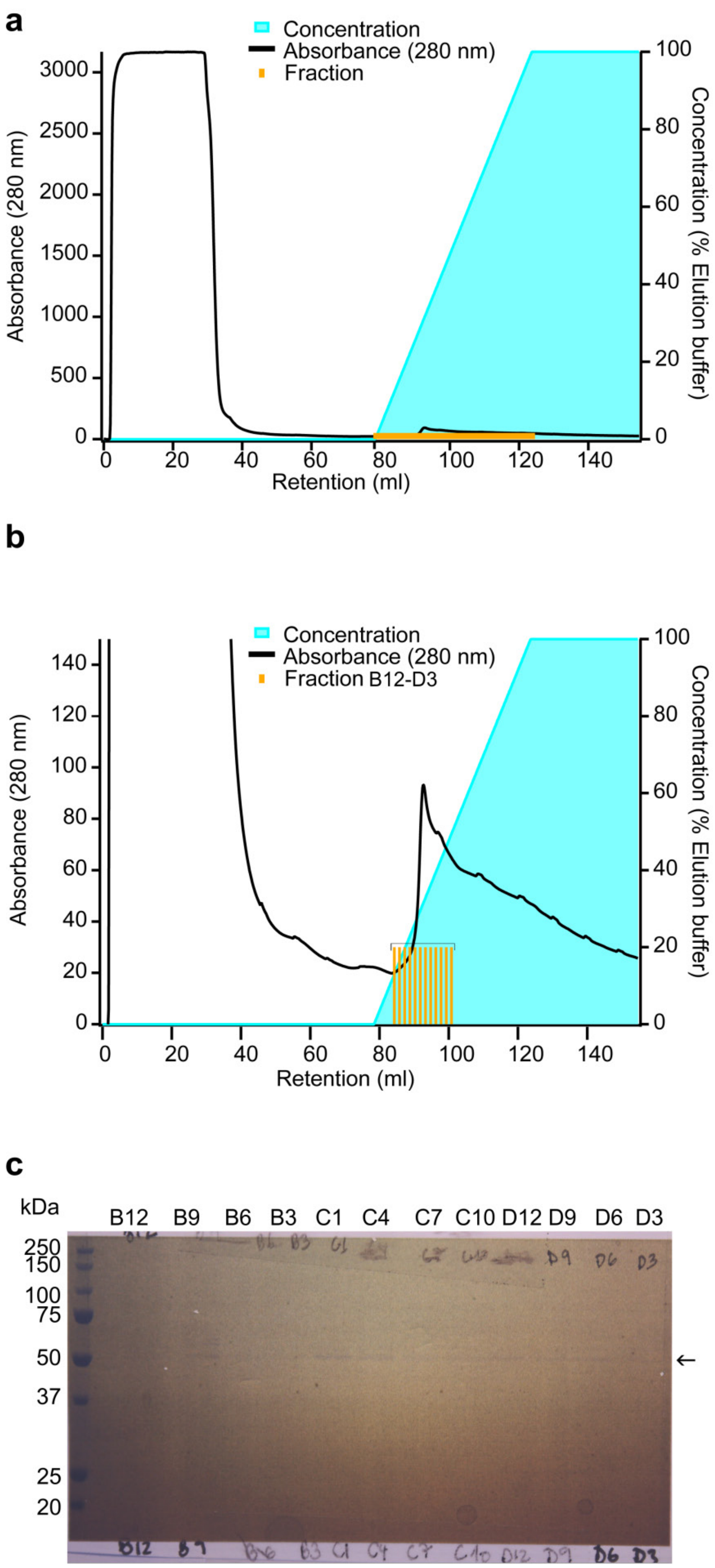

Supplementary Figure 3 | Affinity purification of the DcUGT2-Strep protein Yeast microsomes containing DcUGT2-Strep were solubilized with reduced Triton X-100 and DcUGT2 affinity purified by its Strep-tag II using a *Strep*-Tactin column. **a** The elution profile of DcUGT2-Strep as monitored by the protein absorbance at 280 nm. **b** The elution profile of DcUGT2-Strep as monitored by the protein absorbance at 280 nm. **c** The elution profile of DcUGT2-Strep as monitored by protein absorbance at 280 nm. The scale for absorbance at 280 nm has been adjusted compared to (**a**) to easily distinguish the fractions that were analyzed further. **c** Eluted protein fractions corresponding to the fractions indicated in (**b**) were separated on an SDS gel followed by Coomassie-staining. The arrow points to the position of DcUGT2-Strep.

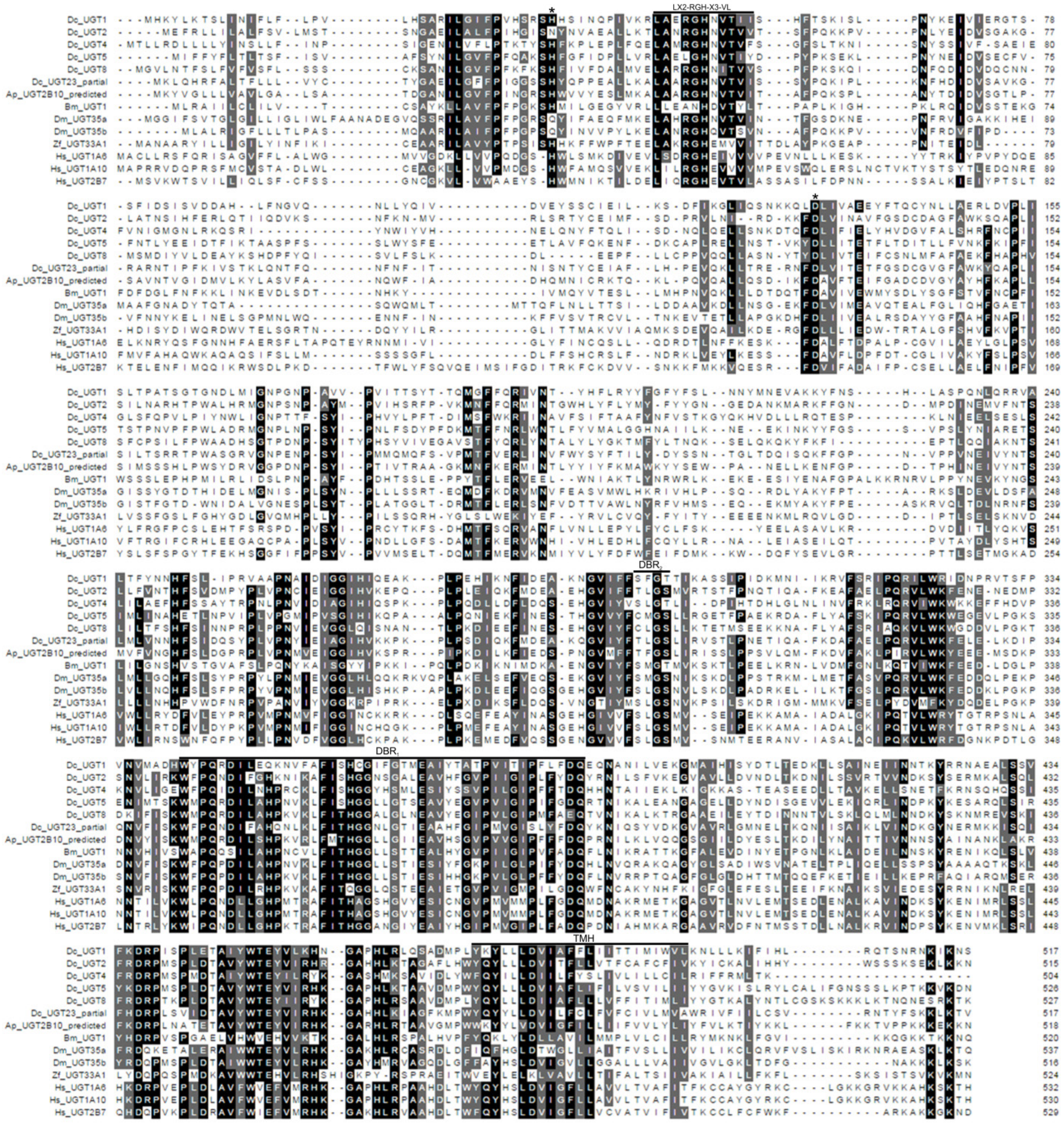

Supplementary Figure 4 | Alignment of UGT amino acid sequences Black and grey indicate identical and similar amino acids, respectively. Multiple sequence alignment was performed with MUSCLE[1] using the MEGA6 software. The alignment is composed of: DcUGT1, DcUGT2, DcUGT4, DcUGT5, DcUGT8 and the partial DcUGT23 from *D. coccus*; the predicted ApUGT2B10 from *A. pisum* (accession no.: XP001948117); BmUGT1 (accession no.: AF324465) from *Bombyx mori*; DmUGT35a (accession no.: AF116555) and DmUGT35b (accession no.: AF116554) from *Drosophila melanogaster*; ZfUGT33A1 (accession no.: GQ915324) from *Zygaena filipendulae*; HsUGT1A6 (accession no.: NM001072), HsUGT1A10 (accession no.: U89508) and HsUGT2B7 (accession no.: NM001074) from *Homo sapiens*. The motifs, “LX2-RGH-X3-VL”, “DBR1” and “DBR_2”, are indicated as described in[2,3]. The predicted transmembrane helix (TMH) is given. Important catalytic residues are indicated with an asterisk according to[2].

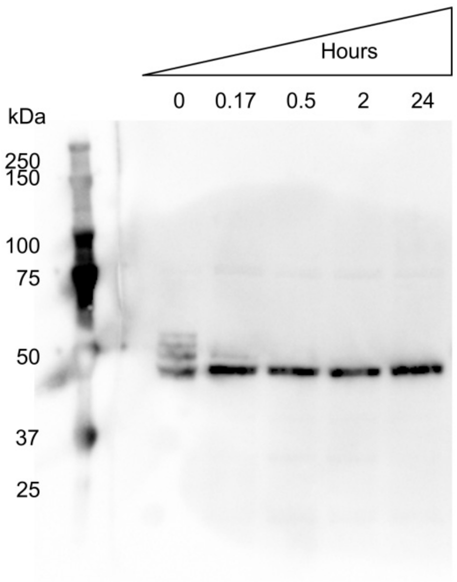

Supplementary Figure 5 | Deglycosylation of truncated DcUGT2ΔMD-Strep protein Yeast microsomes containing DcUGT2ΔMD-Strep were deglycosylated with glycanases for different periods of time: 0 min (0); 10 min (0.17); 30 min (0.5); 2 h (2) and 24 h (24). Proteins were separated on an SDS gel followed by western blotting using an anti-Strep antibody. Note that the anti-strep antibody reacts with the marker protein bands.

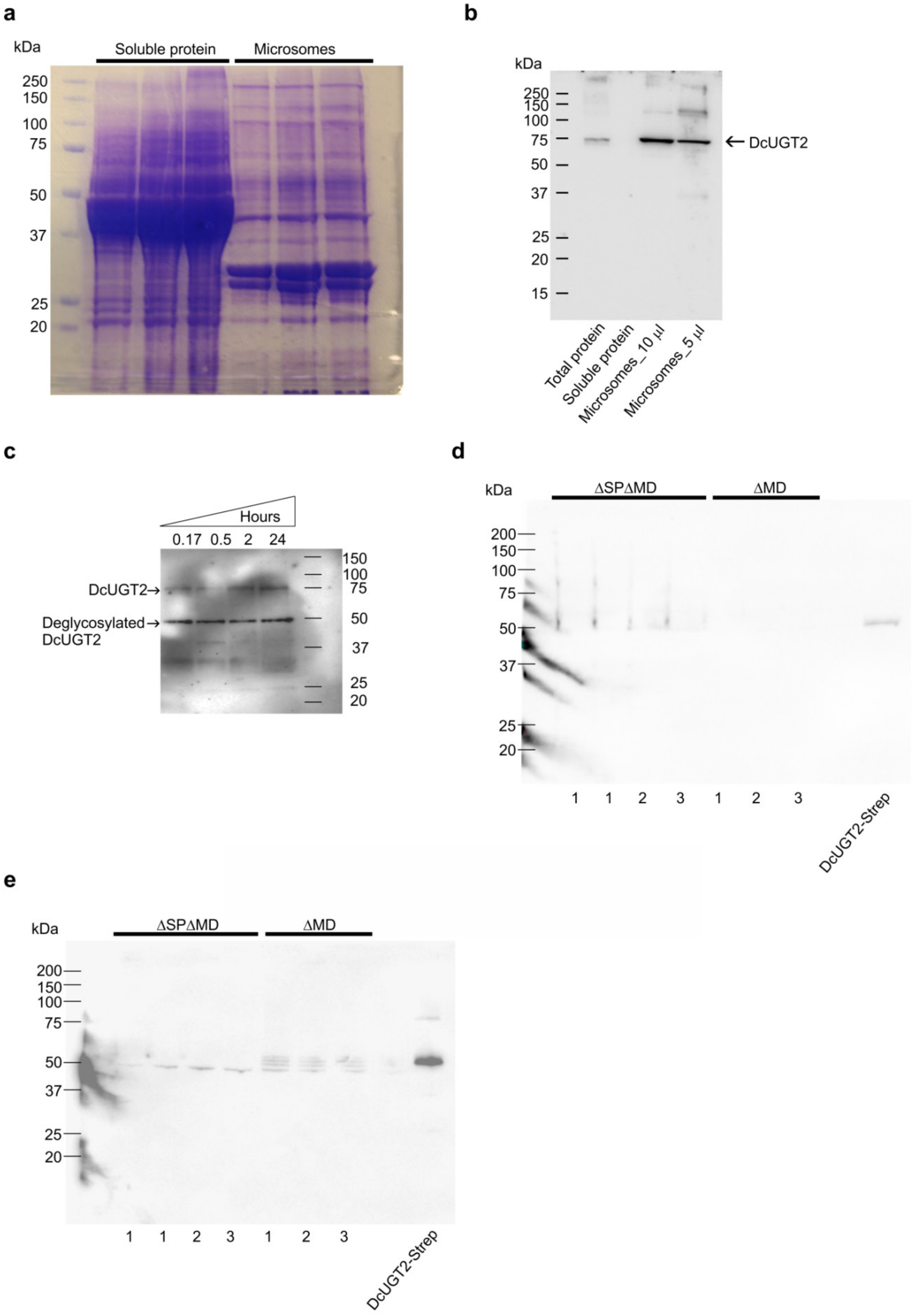

Supplementary Figure 6 | Uncropped gel or blot images Images displayed in cropped versions in the article. **a** Uncropped image of Fig. 2a: Coomassie-stained SDS gel of separated microsomal/soluble protein from *D. coccus*. **b** Uncropped image of Fig. 8a: Non-treated total protein, soluble protein and microsomes from *D. coccus*. Two different amounts of microsomes were loaded. **c** Uncropped image of Fig. 8b: *D. coccus* microsomes treated with glycanases for 10 min (0.17); 30 min (0.5); 2 h (2) and 24 h (24). **d** Uncropped image of Fig. 9a: Soluble protein from three individual *S. cerevisiae* transformants expressing either *ΔSP-DcUGT2ΔMD-Strep* (ΔSPΔMD) or *DcUGT2ΔMD-Strep* (ΔMD) were analyzed by western blotting. Transformant 1 expressing *ΔSP-DcUGT2ΔMD-Strep* was loaded twice. Yeast microsomes, containing the full-length DcUGT2-Strep, were used as an additional size marker control. **e** Uncropped image of Fig. 9b: Microsomal protein from three individual *S. cerevisiae* transformants expressing either *ΔSP-DcUGT2ΔMD-Strep* (ΔSPΔMD) or *DcUGT2ΔMD-Strep* (ΔMD) were analyzed by western blotting. Transformant 1 expressing *ΔSP-DcUGT2ΔMD-Strep* was loaded twice. Yeast microsomes, containing the full-length DcUGT2-Strep, were used as an additional size marker control.

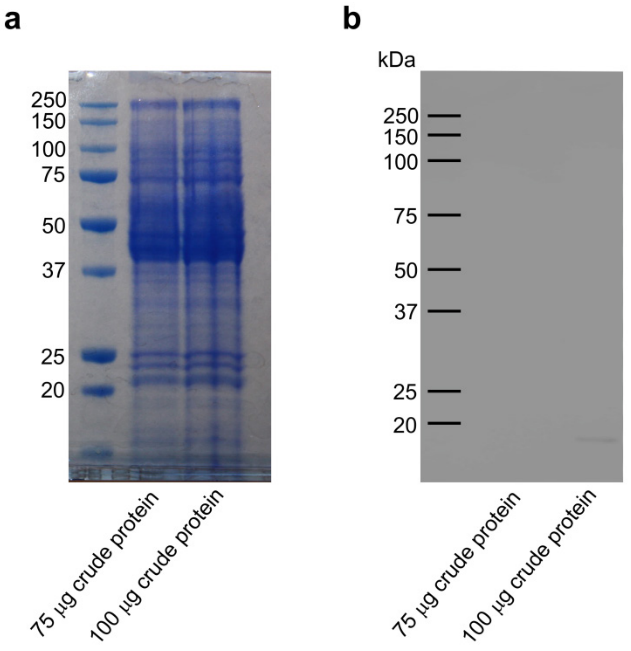

Supplementary Figure 7 |Rabbit pre-immune serum test The pre-immune serum of the rabbit, used to produce anti-DcUGT2 antibodies, was tested for cross-reactivity towards a crude *D. coccus* protein extract. The test was carried out on two amounts (75 µg and 100 µg) of crude protein. **a** Coomassie-stained SDS gel of separated crude *D. coccus* proteins. **b** Western blot analysis of crude *D. coccus* proteins probed with rabbit pre-immune serum (1:1000 dilution), followed by incubation with secondary HRP-conjugated antibodies and chemiluminescence detection.

Supplementary References

1 Edgar, R. C. MUSCLE: multiple sequence alignment with high accuracy and high throughput. *Nucleic Acids Research* **32**, 1792-1797, doi:10.1093/nar/gkh340 (2004).

2 Radominska-Pandya, A., Bratton, S. M., Redinbo, M. R. & Miley, M. J. The crystal structure of human UDP-glucuronosyltransferase 2B7 C-terminal end is the first mammalian UGT target to be revealed: the significance for human UGTs from both the 1A and 2B families. *Drug Metab. Rev.* **42**, 133-144 (2010).

3 Senay, C. *et al.* The importance of cysteine 126 in the human liver UDP-glucuronosyltransferase UGT1A6. *Biochim. Biophys. Acta, Protein Struct. Mol. Enzymol.* **1597**, 90-96 (2002).
