# Supplementary material for: Characterization of a membrane-bound C-glucosyltransferase responsible for carminic acid biosynthesis in Dactylopius coccus Costa
Source: Nat Commun. 2017 Dec 7;8:1987. doi: 10.1038/s41467-017-02031-z (PMC5719414; doi:10.1038/s41467-017-02031-z)
Supplement: Supplementary file 3 — Description of Additional Supplementary Files [file 41467_2017_2031_MOESM3_ESM.pdf]

Description of Additional Supplementary Files

File Name: Supplementary Data 1
Description: **List of putative *UDP-glycosyltransferas*e (*UGT*) candidate transcripts in *Dactylopius coccus.*** UGT candidates were identified based on Pfam annotation and protein homology BLAST analyses. Note that only the transcripts of *DcUGT2*, *DcUGT5* and *DcUGT5* have been verified by RACE. Several of the transcripts might therefore be partial.

File Name: Supplementary Data 2
Description: **Identification of proteins in the *Dactylopius coccus* proteome (protein level, 50-70 kDa region) by searching the *de novo*-assembled *D. coccus* transcriptome database.** Note that "amino acid sequence of the translated verified or predicted ORF" refers to amino acid sequence of the *D. coccus* protein identified based on the *de novo*-assembled transcriptome. Transcript sequences could be partial and have not been verified by RACE (except for *DcUGT2*, *DcUGT4* and *DcUGT5*).

File Name: Supplementary Data 3
Description: **MASCOT generic peak-list file.** The MASCOT generic peak-list file was generated by the Proteome Discoverer software and used to identify proteins in MASCOT searches.
